# Supplementary material for: On the use of kinship and familiarity associated social information in mediating Drosophila melanogaster oviposition decisions
Source: PLoS One. 2025 Mar 26;20(3):e0320377. doi: 10.1371/journal.pone.0320377 (PMC11940635; doi:10.1371/journal.pone.0320377)
Supplement: S1 Figure — b) an observation arena into which the focal ovipositing female was introduced and monitored. At the bottom of the chamber the lids that exhibited different kinds of social information were affixed. c) the arrangement of dishes in the first experiment. In this experiment there were four lids that exhibited social information and the ‘control/unexposed’ dish that contained no social information (as it had no prior exposure to flies). d) the arrangement of the two dishes with social information and the control dish in the second experiment. e) the arrangement of the two dishes with social information and the control dish in the third experiment. f) the arrangement of the two dishes with social information and the control dish in the fourth experiment. (PDF) [file pone.0320377.s001.pdf]

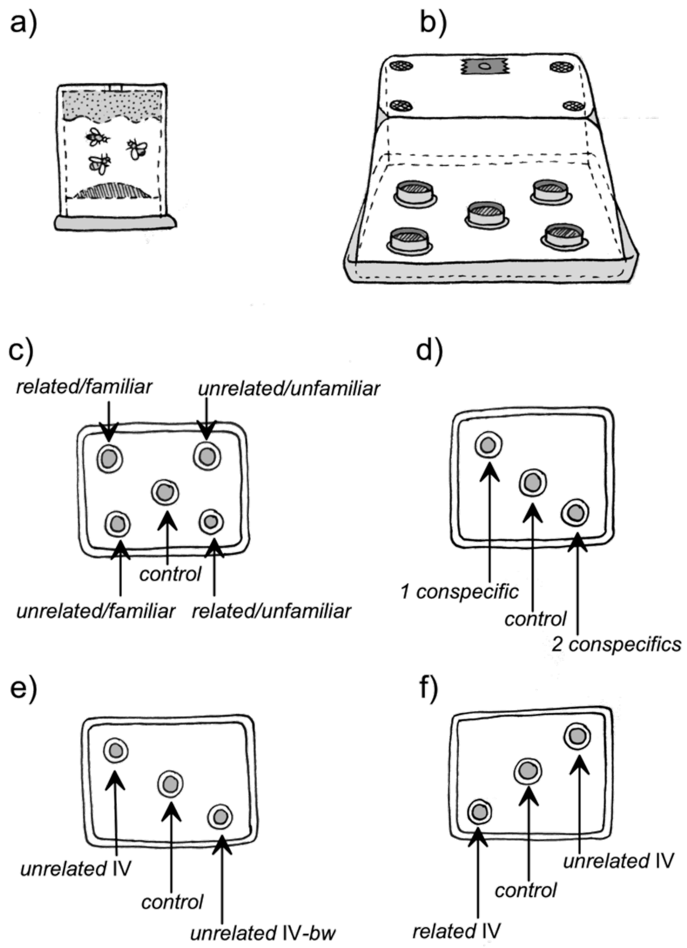

S1 Figure. Drawings illustrating **a)** the "mini-egg" chambers used to collect eggs and/or create media that exhibited social cues. **b)** an observation arena into which the focal ovipositing female was introduced and monitored. At the bottom of the chamber the lids that exhibited different kinds of social information were affixed. **c)** the arrangement of dishes in the first experiment. In this experiment there were four lids that exhibited social information and the 'control/unexposed' dish that contained no social information (as it had no prior exposure to flies). **d)** the arrangement of the two dishes with social information and the control dish in the second experiment. **e)** the arrangement of the two dishes with social information and the control dish in the third experiment. **f)** the arrangement of the two dishes with social information and the control dish in the fourth experiment.
