# Supplementary material for: On the use of kinship and familiarity associated social information in mediating Drosophila melanogaster oviposition decisions
Source: PLoS One. 2025 Mar 26;20(3):e0320377. doi: 10.1371/journal.pone.0320377 (PMC11940635; doi:10.1371/journal.pone.0320377)
Supplement: S2 Figure — Boxplots illustrating the cumulative number of observations made across 26 sessions in the first experiment in which a focal female D.melanogaster was observed on the surface of one of 5 different media dishes present in the 36 replicate arenas. The boxes enclose the middle 50% of data (the inter-quartile range, IQR), with the thick horizontal line representing the location of median. Data points> ± 1.5 * IQR are designated as outliers. Whiskers extend to largest/smallest values that are not outliers, indicated as closed circles. The results of a Tukey HSD post-hoc test comparing group mean is indicated by letters, where groups that do not share the same letter are considered statistically different at the α=0.05 level. (PDF) [file pone.0320377.s002.pdf]

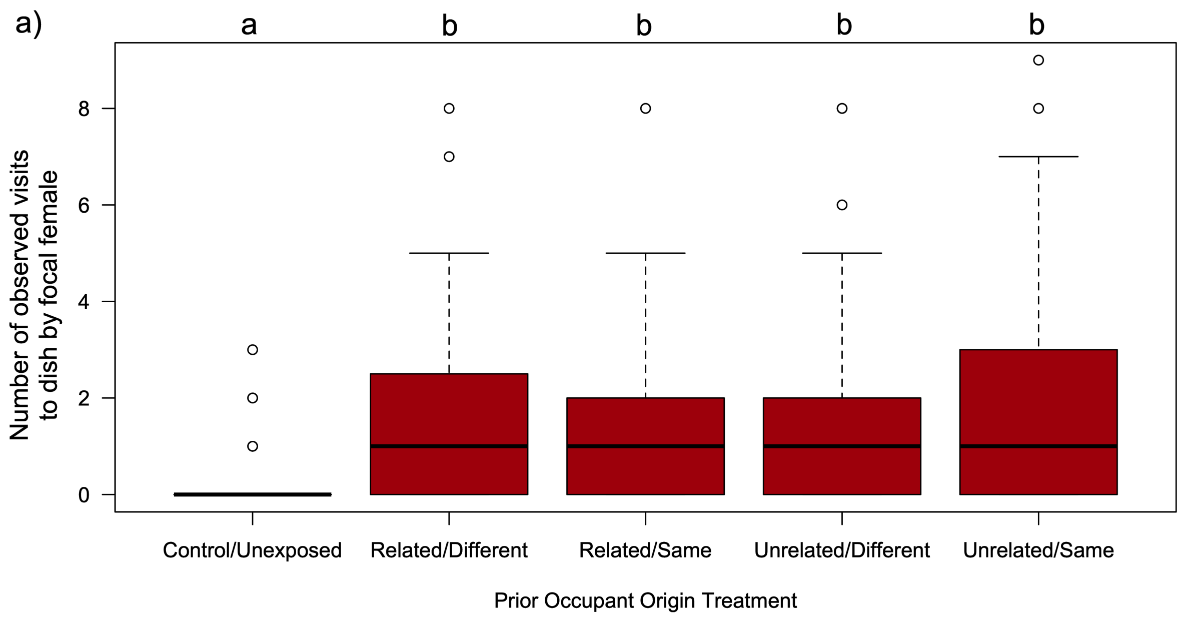

S2 Figure. Focal females visited demonstrator-exposed dishes more frequently than to control/unexposed dishes. Boxplots illustrating the cumulative number of observations made across 26 sessions in the first experiment in which a focal female *D. melanogaster* was observed on the surface of one of 5 different media dishes present in the 36 replicate arenas. The boxes enclose the middle 50% of data (the inter-quartile range, IQR), with the thick horizontal line representing the location of median. Data points > ±1.5*IQR are designated as outliers. Whiskers extend to largest/smallest values that are not outliers, indicated as closed circles. The results of a Tukey HSD post-hoc test comparing group mean is indicated by letters, where groups that do not share the same letter are considered statistically different at the α=0.05 level.
