# Supplementary material for: On the use of kinship and familiarity associated social information in mediating Drosophila melanogaster oviposition decisions
Source: PLoS One. 2025 Mar 26;20(3):e0320377. doi: 10.1371/journal.pone.0320377 (PMC11940635; doi:10.1371/journal.pone.0320377)
Supplement: S4 Figure — Boxplots illustrating the cumulative number of observations made across 26 sessions in the fourth experiment in which a focal female D. melanogaster was observed on the surface of one of 3 different media dishes present in the 84 replicate arenas that had previously been exposed to either a related IV female (mated to a IVbwD male), an unrelated IV female (mated to a IV-bwD male), or neither. Boxplot components as in Figure S2. The results of a Tukey HSD post-hoc test comparing group mean is indicated by letters, where groups that do not share the same letter are considered statistically different at the α=0.05 level. (PDF) [file pone.0320377.s004.pdf]

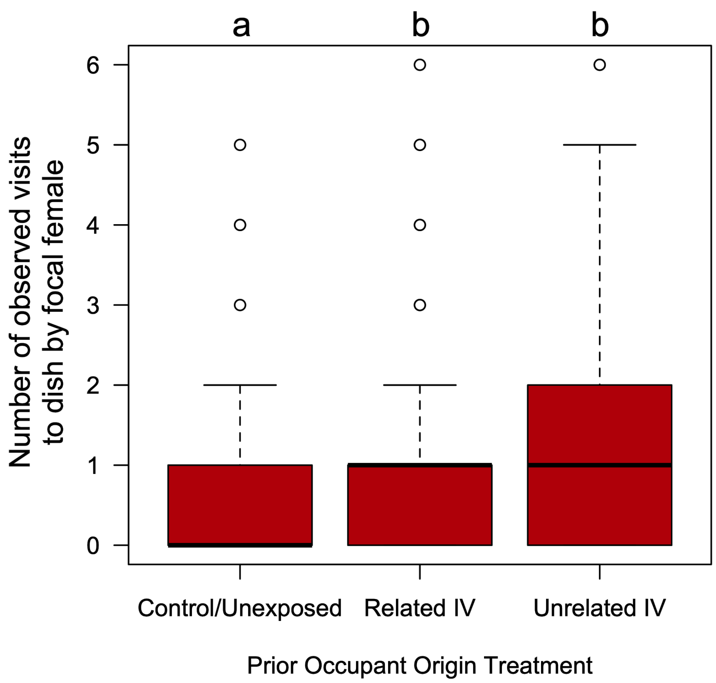

S4 Figure. Focal females visited demonstrator-exposed dishes more frequently than to control/unexposed dishes. Boxplots illustrating the cumulative number of observations made across 26 sessions in the fourth experiment in which a focal female *D. melanogaster* was observed on the surface of one of 3 different media dishes present in the 84 replicate arenas that had previously been exposed to either a related IV female (mated to a IV-*bwD* male), an unrelated IV female (mated to a IV-*bwD* male), or neither. Boxplot components as in Figure S2. The results of a Tukey HSD post-hoc test comparing group mean is indicated by letters, where groups that do not share the same letter are considered statistically different at the $\alpha=0.05$ level.
