# Supplementary material for: On the use of kinship and familiarity associated social information in mediating Drosophila melanogaster oviposition decisions
Source: PLoS One. 2025 Mar 26;20(3):e0320377. doi: 10.1371/journal.pone.0320377 (PMC11940635; doi:10.1371/journal.pone.0320377)
Supplement: S5 Figure — Boxplots illustrating the number of brown-eyed adult flies that eclosed from dishes that had been exposed to either a related IV female (mated to a IV-bwD male), an unrelated IV female (mated to a IV-bwD male), or neither in the fourth experiment. There was a small (Cliff’s Delta effect size = 0.15) but statistically significant difference in the mean number of offspring collected from these dishes. Boxplot components as in Figure S2. The control/unexposed dishes my definition had not had any prior exposure to a female capable of producing brown-eyed offspring, and thus those dishes never yielded any offspring with that phenotype. (PDF) [file pone.0320377.s005.pdf]

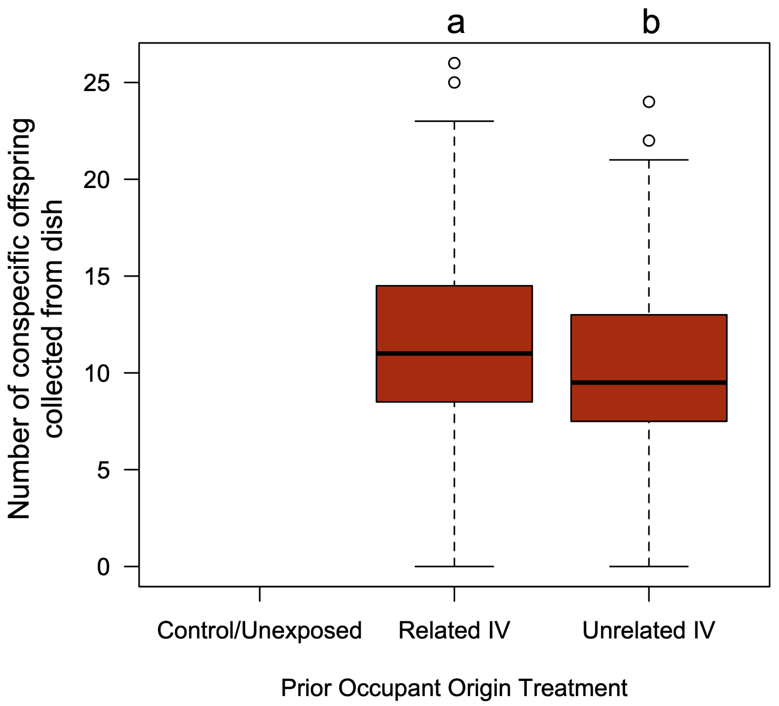

S5 Figure. Number of conspecific offspring collected from demonstrator-exposed dishes. Boxplots illustrating the number of *brown-eyed* adult flies that eclosed from dishes that had been exposed to either a related IV female (mated to a IV-*bwD* male), an unrelated IV female (mated to a IV-*bwD* male), or neither in the fourth experiment. There was a small (Cliff's Delta effect size=0.15) but statistically significant difference in the mean number of offspring collected from these dishes. Boxplot components as in Figure S2. The control/unexposed dishes my definition had not had any prior exposure to a female capable of producing *brown-eyed* offspring, and thus those dishes never yielded any offspring with that phenotype.
