# Supplementary material for: On the use of kinship and familiarity associated social information in mediating Drosophila melanogaster oviposition decisions
Source: PLoS One. 2025 Mar 26;20(3):e0320377. doi: 10.1371/journal.pone.0320377 (PMC11940635; doi:10.1371/journal.pone.0320377)
Supplement: S6 Figure — While both subset means are the same, as there is a greater number of IV-bw eggs in the vial than IV eggs, the larger sample size of the former will likely contain individuals exhibiting a wider range of phenotypes than the latter. The unanticipated consequence of this being that when sampling for virgin female flies, there is a greater probability of collecting a suitable IV-bw before encountering a suitable IV female. As body size is negatively correlated with eclosion time (Partridge et al. 1987), and positively correlated with female fecundity (Lefranc and Bundgaard 2000, Long et al. 2009), it is conceivable that the IV-bw and the IV females collected and used as demonstrators differed in their capacity to produce offspring, resulting in the conspecific patterns seen in Figure 1b and 3b. It is worth noting that in our fourth experiment, where only red-eyed females were collected from vials containing both IV and IV-bw eggs that there was only a small difference in the number of demonstrator offspring that were collected (Figure S5), which is consistent with the hypothesis described above. (PDF) [file pone.0320377.s006.pdf]

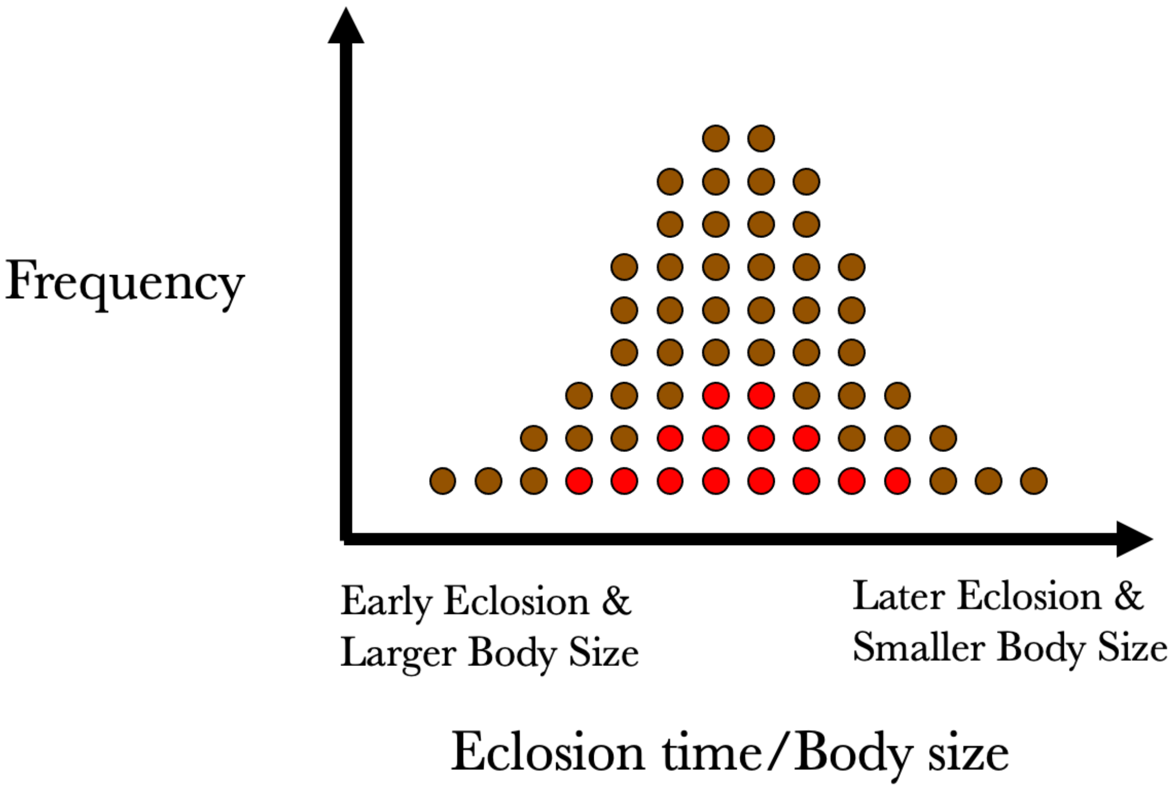

S6 Figure. Schematic diagram depicting hypothetical distribution of female *D. melanogaster* eclosion times in a mixture of eggs collected at the same time from the IV and the IV-*bw* populations (where we assume are randomly sampled, and that both populations have identical distributions, means and variances). While both subset means are the same, as there is a greater number of IV-*bw* eggs in the vial than IV eggs, the larger sample size of the former will likely contain individuals exhibiting a wider range of phenotypes than the latter. The unanticipated consequence of this being that when sampling for virgin female flies, there is a greater probability of colleting a suitable IV-*bw* before encountering a suitable IV female. As body size is negatively correlated with eclosion time (Partridge et al. 1987), and positively correlated with female fecundity (Lefranc and Bundgaard 2000, Long et al. 2009), it is conceivable that the IV-*bw* and the IV females collected and used as demonstrators differed in their capacity to produce offspring, resulting in the conspecific patterns seen in Figure 1b and 3b. It is worth noting that in our fourth experiment, where only *red-eyed* females were collected from vials containing both IV and IV-*bw* eggs that there was only a small difference in the number of demonstrator offspring that were collected (Figure S5), which is consistent with the hypothesis described above.
