# Supplementary material for: On the use of kinship and familiarity associated social information in mediating Drosophila melanogaster oviposition decisions
Source: PLoS One. 2025 Mar 26;20(3):e0320377. doi: 10.1371/journal.pone.0320377 (PMC11940635; doi:10.1371/journal.pone.0320377)
Supplement: Attachment — Submitted filename: Response to Reviewers.docx [file pone.0320377.s007.docx]

Sent: Wednesday, October 2, 2024 9:33 PM

To: Emily Rakosy <emily.rakosy@mail.utoronto.ca>

Subject: PLOS ONE Decision: Revision required [PONE-D-24-34027] - [EMID:9284e0c42c80af60]

PONE-D-24-34027

On the use of kinship and familiarity associated social information in mediating Drosophila melanogaster oviposition decisions

PLOS ONE

Dear Dr. Rakosy,

Thank you for submitting your manuscript to PLOS ONE. After careful consideration, we feel that it has merit but does not fully meet PLOS ONE’s publication criteria as it currently stands. Therefore, we invite you to submit a revised version of the manuscript that addresses the points raised during the review process.

**We thank the editors and reviewers for their thoughtful feedback and comments. Below list how and where we were able to respond to these suggestions.**

==============================

The reviewer's reports are included at the bottom of this email or can be accessed, together with a copy of this decision letter, by logging on to the journal editorial manager.

As you will note, the reviewers raise a number of substantial criticisms that prevent me from accepting the manuscript at this stage. Both reviewer's solicit additional clarification on certain outstanding issues including experimental rationale, sample number, data analysis, etc., and provide constructive feedback to improve the clarity and presentation of the article. Additionally, we note that the online repository link for deposited data is not working.

==============================

Please submit your revised manuscript by Nov 16 2024 11:59PM. If you will need more time than this to complete your revisions, please reply to this message or contact the journal office at plosone@plos.org. When you're ready to submit your revision, log on to https://www.editorialmanager.com/pone/ and select the 'Submissions Needing Revision' folder to locate your manuscript file.

Please include the following items when submitting your revised manuscript:

A rebuttal letter that responds to each point raised by the academic editor and reviewer(s). You should upload this letter as a separate file labeled 'Response to Reviewers'.

A marked-up copy of your manuscript that highlights changes made to the original version. You should upload this as a separate file labeled 'Revised Manuscript with Track Changes'.

An unmarked version of your revised paper without tracked changes. You should upload this as a separate file labeled 'Manuscript'.

If you would like to make changes to your financial disclosure, please include your updated statement in your cover letter. Guidelines for resubmitting your figure files are available below the reviewer comments at the end of this letter.

If applicable, we recommend that you deposit your laboratory protocols in protocols.io to enhance the reproducibility of your results. Protocols.io assigns your protocol its own identifier (DOI) so that it can be cited independently in the future. For instructions see: https://journals.plos.org/plosone/s/submission-guidelines#loc-laboratory-protocols. Additionally, PLOS ONE offers an option for publishing peer-reviewed Lab Protocol articles, which describe protocols hosted on protocols.io. Read more information on sharing protocols at https://plos.org/protocols?utm_medium=editorial-email&utm_source=authorletters&utm_campaign=protocols.

We look forward to receiving your revised manuscript.

Kind regards,

Madhumala Sadanandappa, PhD

Academic Editor

PLOS ONE

Journal Requirements:

When submitting your revision, we need you to address these additional requirements.

1. Please ensure that your manuscript meets PLOS ONE's style requirements, including those for file naming. The PLOS ONE style templates can be found at

https://journals.plos.org/plosone/s/file?id=wjVg/PLOSOne_formatting_sample_main_body.pdf and

https://journals.plos.org/plosone/s/file?id=ba62/PLOSOne_formatting_sample_title_authors_affiliations.pdf

**We have reviewed these these style requirements, and have edited the document in the manner specified**

2. Please update your submission to use the PLOS LaTeX template. The template and more information on our requirements for LaTeX submissions can be found at <http://journals.plos.org/plosone/s/latex>.

**This manuscript was not written in LaTex.**

3. Thank you for stating the following in the Acknowledgments Section of your manuscript:

"We would like to thank Simran Mann, Lukas Ghiglione, and Harleen Taneja of the #DrosLife Lab for their fly-pushing, behavioral observations and camaraderie. Natasha B. Gallo provided helpful feed-back and constructive comments. Ruby Lindsay and Michael Steeleworthy of the Wilfrid Laurier University Library are thanked for their help with data archiving. T.A.F.L. was funded with a Natural Sciences and Engineering Research Council Discovery grants (RGPIN740 2016-06133 and RGPIN-2022-03988). This work was conducted at Wilfrid Laurier University, which exists on the traditional territory of the Neutral, Anishnawbe, and Haudenosaunee peoples."

We note that you have provided funding information that is not currently declared in your Funding Statement. However, funding information should not appear in the Acknowledgments section or other areas of your manuscript. We will only publish funding information present in the Funding Statement section of the online submission form.

Please remove any funding-related text from the manuscript and let us know how you would like to update your Funding Statement. Currently, your Funding Statement reads as follows:

"TAFL received support in the for of Natural Sciences and Engineering Research Council (https://www.nserc-crsng.gc.ca) Discovery grants (RGPIN-2016-06133 and RGPIN-2022-03988). The funding agency played no role in the study design, data collection and analysis, decision to publish, or preparation of the manuscript"

Please include your amended statements within your cover letter; we will change the online submission form on your behalf.

We have removed referenes to the funding sources in the acknowledgements. The amended statements are as follows

**Acknowledgements**

**We would like to thank Simran Mann, Lukas Ghiglione, and Harleen Taneja of the #DrosLife Lab for their fly-pushing, behavioral observations and camaraderie. Natasha B. Gallo and two anonymous reviewers provided helpful feed-back and constructive comments. Ruby Lindsay and Michael Steeleworthy of the Wilfrid Laurier University Library are thanked for their help with data archiving. This work was conducted at Wilfrid Laurier University, which exists on the traditional territory of the Neutral, Anishnawbe, and Haudenosaunee peoples.**

**Funding Statement**

**TAFL received support in the for of Natural Sciences and Engineering Research Council (https://www.nserc-crsng.gc.ca) Discovery grants (RGPIN-2016-06133 and RGPIN-2022-03988). The funding agency played no role in the study design, data collection and analysis, decision to publish, or preparation of the manuscript**

4. Please note that in order to use the direct billing option the corresponding author must be affiliated with the chosen institute. Please either amend your manuscript to change the affiliation or corresponding author, or email us at plosone@plos.org with a request to remove this option.

**The corresponding autor (TAFL) is affiliated with the chosen institute (Wilfrid Laurier University)**

5. We notice that your supplementary figures are included in the manuscript file. Please remove them and upload them with the file type 'Supporting Information'. Please ensure that each Supporting Information file has a legend listed in the manuscript after the references list.

**We have removed the supplementary figures from the manuscript, and have uploaded them as Supporting Information.**

6. Please review your reference list to ensure that it is complete and correct. If you have cited papers that have been retracted, please include the rationale for doing so in the manuscript text, or remove these references and replace them with relevant current references. Any changes to the reference list should be mentioned in the rebuttal letter that accompanies your revised manuscript. If you need to cite a retracted article, indicate the article’s retracted status in the References list and also include a citation and full reference for the retraction notice.

**The reference list has been reviewed to ensure it is complete and correct.**

[Note: HTML markup is below. Please do not edit.]

Reviewers' comments:

Reviewer's Responses to Questions

Comments to the Author

1. Is the manuscript technically sound, and do the data support the conclusions?

The manuscript must describe a technically sound piece of scientific research with data that supports the conclusions. Experiments must have been conducted rigorously, with appropriate controls, replication, and sample sizes. The conclusions must be drawn appropriately based on the data presented.

Reviewer #1: Yes

Reviewer #2: Yes

2. Has the statistical analysis been performed appropriately and rigorously?

Reviewer #1: Yes

Reviewer #2: Yes

3. Have the authors made all data underlying the findings in their manuscript fully available?

The PLOS Data policy requires authors to make all data underlying the findings described in their manuscript fully available without restriction, with rare exception (please refer to the Data Availability Statement in the manuscript PDF file). The data should be provided as part of the manuscript or its supporting information, or deposited to a public repository. For example, in addition to summary statistics, the data points behind means, medians and variance measures should be available. If there are restrictions on publicly sharing data—e.g. participant privacy or use of data from a third party—those must be specified.

Reviewer #1: No

Reviewer #2: Yes

4. Is the manuscript presented in an intelligible fashion and written in standard English?

PLOS ONE does not copyedit accepted manuscripts, so the language in submitted articles must be clear, correct, and unambiguous. Any typographical or grammatical errors should be corrected at revision, so please note any specific errors here.

Reviewer #1: Yes

Reviewer #2: Yes

5. Review Comments to the Author

Please use the space provided to explain your answers to the questions above. You may also include additional comments for the author, including concerns about dual publication, research ethics, or publication ethics. (Please upload your review as an attachment if it exceeds 20,000 characters)

Reviewer #1: I really enjoyed reading this paper – I thought it was clearly written and the results support the conclusions. The authors address the interesting question of whether relatedness of conspecific females plays a role in how Drosophila use public information to inform egg laying decisions. This is a novel study, which finds some evidence that relatedness and population of origin of conspecifics are likely important factors in female patch preferences. The authors also try to separate out some confounding factors of their main experiment. These latter assays do somewhat strengthen the hypothesis that relatedness is important, but further follow up experiments are probably required to strongly conclude that females are responding to relatedness of conspecifics over other factors. However, I don’t think the authors overstate their results in their conclusions, and I am happy that the study meets the requirements for publication in PLosOne.

**We are very happy to hear that the reviewer enjoyed reading this paper, and thank them for their comments and suggetions!**

I have some suggested minor revisions:

Sample sizes: Apologies if I missed it, but I can’t see any sample sizes for the different experiments? I see from Experiment 1 that there were 50 families created, but not clear if that means n = 50 for that experiment? And not sure for the other three experiments.

It might be personal preference, but I also find it useful when all data points are visible on the boxplots to give an instant idea of sample size. This is not as essential as stating the sample sizes clearly somewhere in the text though.

**Thank you for the helpful suggestion. We have updated our methods section to (lines 254, 280, 299, 320) so that the sample sizes are easily available. We have also added the sample sizes to all the figure legends (Figures 1-4, S2-24) for the benefit of the reader.**

Throughout the manuscript, authors refer to “egg-laying” patterns or number of eggs laid, but as far as I can tell, they actually measure adult offspring (e.g. lines 264-267 imply that egg numbers are being counted). I understand that offspring is likely a good proxy for number of eggs laid, but egg-adult viability can be <100% and might differ between treatments (e.g. density on a patch can affect developmental success). I think the authors could consider using more specific language (i.e. adult offspring, not eggs), unless they did any egg counts to verify viability? Authors could add a discussion point around using number of eclosed adult offspring as a proxy for egg number.

**This is an important point. In our population there is high egg-to-adult survivorship in vials of moderate to low larval densities. We have specifically addressed this decision and our justification on Line 266-268: “**We used the number of eclosed adult offspring as a proxy for the egg number laid, in this and subsequent experiments, as in the IV population at moderate larval densities, there is very high egg-to-adult survivorship (Khodaei et al. 2020).”

A similar point applies to axis titles etc. “number of conspecific offspring already on patch” is not what is measured (I think). It is an assumption that the number of adult offspring that belong to the demonstrator female was equal to the number of eggs on the dish encountered by the focal female. There can always be more eggs or larvae than adult offspring.

**We have revised all the relevant figures (Figures 1, 2, 3 4, S5) to state that the y-axis depicts the number of conspeficic/focal offspring collected from the dish to better describe our measured variable and the underlying assumption.**

Line 405-407: I think there is a typo here – one of the dishes contains a related female, right?

**Thank you. We have corrected (this embarrassing) typo (Line 447-450: “**In our fourth experiment, we set out (again) to compare the patterns of dish visitation and offspring production by focal females on three different dishes (a control/unexposed dish, a dish whose demonstrator was a related IV female, or a dish where the demonstrator was an unrelated IV female)”

Line 358: this should refer to Figure 1a specifically, then Figure 1b should be referenced in the text somewhere (lines 363-65?) but isn’t currently.

**We have revised this section to specifically mention Figure 1a and 1b individually. (Lines 378 & 391)**

Line 363-65: “more brown-eyed offspring eclosing from vials whose prior occupant was an unrelated female (IV-bw, mated to a IV-bwD male) than those that had previously been exposed to an unrelated female” Possible typo - should this read related?

**You are correct! We have corrected this typo (Lines 393-395) “**with more brown-eyed offspring eclosing from dishes whose prior occupant was a unrelated female (IV-*bw*, mated to a IV-*bwD* male; x̄=14.49; 95% CI: 13.03, 16.10) than those that had previously been exposed to a related female (IV, mated to a IV-*bwD* male; x̄=6.83; 95% CI: 5.95, 7.83; LRχ^2^ =147.93, df=1, p=4.90x10^-34^)”

Line 457-463: I think this sentence could be broken down into 2-3 sentences, it was quite hard to read.

**Thank you for the suggestion. We agree that this is a tricky passage, and have broken it up, and reworded it so that it is hopefully easier to read (Lines 507-515): “**Interestingly, Malek and Long (2020) observed that a focal female’s offspring production patterns depended on the developmental environment of *both* the focal female and the demonstrator. In one assay, they observed focal females who developed in a protein-rich vial environment, oviposited more on dishes where the demonstrator females (that came from the same population) who had also developed on similar protein-rich media. However, in a second assay when the focal females developed on standard media, these flies laid fewer eggs on dishes where the demonstrator came from the same population, but had instead developed on protein-rich media, than they did on dishes where the demonstrator came from a different *D. melanogaster* population but had *also* developed on standard media.”

Reviewer #2: Dear editor,

Please find included below my review for manuscript PONE-D-24-34027, “On the use of kinship and familiarity associated social information in mediating Drosophila melanogaster oviposition decisions”, by Emily Rakosy, Sanduni Talagala and Tristan A.F. Long. In this manuscript, the authors present a study on the use of social information on oviposition behaviour in D. melanogaster. In this species, mothers can profoundly affect the fitness of their offspring by deciding to oviposit in patches that may or may not be suitable. Determining whether a patch is suitable may be time- or resource-consuming. As a workaround, females may make use of social information about patches to decide to oviposit or not. In particular, cues left by related females may signal that a patch is particularly favourable, owing to e.g. reduced competition among developing kin and intraspecific variation for ability to use specific resources. The authors thus set out to test the hypothesis that D. melanogaster females prefer to oviposit with related females. To do so, they use a series of choice experiments in which they expose focal females to oviposition substrate previously used by genetic kin and non-kin, in addition to varying the developmental background in which these prior ovipositors develop (which might disrupt kin recognition). They find that focal females prefer to oviposit on patches previously used by related females, provided that they both grew up in a similar environment.

Overall, I think the work done is interesting, and the approach is suitable. I do, however, think that the paper can be revised to be clearer. There are various bits where the authors present information that I think would be better off elsewhere, which to me feels like things are not in the right order – though I’ll also admit this is (partially) a matter of taste.

**We thank the reviewer for their helpful comments, that led us to rethink many parts of the analysis and structuring of the manuscript. We hope that these changes are to their satisfaction.**

Major comments:

Line 144-178: This feels like a disproportionate amount of text devoted to discussing the way CHCs may be used to obtain social information given that there is no direct test of how CHCs may be used by females. Admittedly, the different development backgrounds used in the experiments might help reveal such effects, but this is still a rather indirect measure. I think the length of this section can be reduced (but see next comment).

**We thank the reviewer for their comment. Upon review (and especially in conjunction with the reviewer’s next comment), we have tried to tighten up this section (Lines 127-136). However, we have retained most of the CHC material, as we do want to inspire others to explore the specific role played these molecules (and/or microbiotic cues), and did not want to be vague about what we suspect the kinship cues may be (and the complications involved)**

Line 154-159: This seems like an important detail for the experimental setup, but I don’t think this is sufficiently clear. Do you mean to say that kin signals are only reliable/recognizable if both individuals developed in a similar background? Or is it purely this background that is being detected, which implies kin due to oviposition sites being patchy? In the case of the latter, wouldn’t such muddying effects make “kin recognition” unreliable? This seems particularly important if there is indeed a lot of intraspecific genetic variation that can affect performance on different substrates (lines 122-125). I think this is the critical bit in this whole section (see previous comment) that I think needs to be made clearer. Reducing the amount of text devoted to the remainder of this section should free up some space to clarify this further.

**Based on the reviewers comments, we have re-written this section to highlight the complicated nature of the factors that could affect cues of kinship. One of the reasons we did lean into the review of CHCs/microbiomes, and the potentially interacting effects with developmental environment, is to highlight that there currently is no clear conclusion that can be drawn from previous work whether kinship recognition is only based on true genetic relatedness, is inferred from environmental cues of familiarity or depends on both. It sets up our experimental justification for manipulating both of these factors in our experiments, so that if we detect biases we can make educated guesses at what cues the flies are using (and if they provide accurate information about relatedness). We agree this is a critical point, and hope that our revisions makes this clear to the reader. See Lines 146-153**

An important and potentially complicating factor is that CHC phenotypes and/or microbiotic communities in *D. melanogaster* can also be influenced by the flies’ developmental environment (Sharon et al. 2010, Fedina et al. 2012, Lizé et al. 2014, Venu et al. 2014, Heys et al. 2018, García‐Roa et al. 2022, Pavković‐Lučić et al. 2022). This raises the possibility that the phenomenon of “kin recognition” may be based on inherited cues of relatedness, on the recognition of similarity of environmental histories (“familiarity”), or some combination of these two factors, and that furthermore under some circumstances that these cues might not provide contradictory or unreliable information about kinship.

**And lines 169-175**

Together these studies highlight that socially-available kinship cues (or proxies thereof) may involve a number of independent and inter-related olfactory signals, whose expression may depend not only on relatedness but also on the specific nature of the developmental environment. As such, any study – such as ours – that explores the potential role of kin recognition in decision making, must explore how individuals respond to cues produced by demonstrators in which relatedness and familiarity are independently manipulated.

Line 179-185: Could you be a bit more explicit about what you test? I know this comes in the next section / next few paragraphs on the experiments, but in its current form this feels perfunctory.

**We have expanded these lines to be more explicit about what we manipulated and measured. Lines 176-184:**

With these ideas and findings in mind, in this study we set out to examine whether female flies showed bias in their use of social information cues left behind by conspecifics when selecting ovipositing sites, and would differ their use of egg-laying substrates that had previously been in contact with either kin or non-kin demonstrator females. Simultaneously, we also explored whether a female’s response to potential kinship cues were conditional on the conspecifics’ “familiarity”, by concurrently manipulating the developmental environment from which the demonstrator females were obtained. Overall, the goal of this research is to better characterize copying behavior in *D. melanogaster* and to gain better insight into the factors that influence these oviposition site decisions.

Regarding the data analysis, I have several questions/concerns.

Line 318-320: The authors imply here that they analyse the number of visits made by each female. In this case, the model setup here would be inadequate, in that this would be better modelled as a mixed-effect binomial GLM (something along the lines of visits ~ lid type + (1 | ID), where the last term describes the random effect of female ID on the probability of visits). This would actually apply to the data in the supplementary figures S2-S4. In addition, I wonder if it might be better to report the analyses for the main results first, and then these analyses that are in the supplementary material. This at least threw me off a bit when I was reading through the paper for the first time.

The data in Figures 1-4 actually show data on the eggs laid on each lid type. These are probably adequately modelled using a Poisson-based model. But looking at the type of data, I would argue that a zero-inflated Poisson model might actually be better (or alternatively, a zero-inflated negative binomial model, depending on variance in the data). It might be worth fitting some extra models to figure out which one best explains the observed data.

But looking at the data again, I wonder whether I am interpreting it correctly – are these really offspring counts, and not counts of females that have laid any eggs whatsoever on the different lid types? The numbers seem quite low for a D. melanogaster female. Or is that because the egg-laying substrate here is so small, and only supports a small number of developing offspring? It might be good to explain these low offspring counts somewhere, and perhaps even explicitly mention something like “females typically produce 3-7 offspring per lid” so people have some extra confirmation that this is actually the case.

**We thank the reviewer for these helpful comments and suggestions. We have redone all of our behavioural and offspring analyses as a result by running zero-inflated generalized mixed models with binomial (for visitation rates) or poisson (for offspring counts) error rates, as these are more appropriate statistically and provide better fits that the GLMs we had originally run. For the most part, our statistical conclusions remain unchanged.**

**After great consideration, we have kept the order in which the results are presented and discussed in the original order (organized by experiments, with alternating references to the main text figures and the supplemental figures) as it matched the actual order in which the data were collected, and, in our opinion, results in a cohesiveness of the discussion of each experiment’s results that does not happen if they are re-organized by finding type.**

**We agree that the offspring yields do appear somewhat low. This may be a side-effect of the use of the larger arenas, where flies may be spending more time moving around between sites (compared to in a vial where there is only one available substrate for oviposition) or (more likely) that fruit flies are social creatures, where socially-isolated females are more inhibited to lay their eggs (see Bailly et al. 2023, Current Biology** [**https://www.cell.com/current-biology/fulltext/S0960-9822(23)00750-9**](https://www.cell.com/current-biology/fulltext/S0960-9822(23)00750-9) **and Churchill et al. 2024 *bioRxiv*** [**https://www.biorxiv.org/content/10.1101/2024.07.03.600353v1.abstract**](https://www.biorxiv.org/content/10.1101/2024.07.03.600353v1.abstract)**). We had considered placing more flies into each chamber, but in that case one could not tell if the focal females were using cues produced by the previous demonstrators, or copying each other.**

Figure captions: All of them start with “boxplot illustrating data collected in the Xth experiment of […]”. I think it would be better to write something in the form of a conclusion as a title. Furthermore, while the description of the various boxplot elements may be informative (I think this excessive, but that is a matter of taste), I find that many of the figure captions are not very informative about what the data actually is and shows.

**Based on this suggestion, we have added a sentence at the start of each figure caption that summarizes the main result being depicted in each figure.**

Minor comments:

Line 14: The abstract feels very long to me, but I’m not sure what the policy here is exactly. Regardless, I think the abstract can be written in a more direct manner.

**We apologize that the abstract feels long- with four inter-connected studies, we may have gone a bit overboard. We have edited the abstract to bring it down below PLoS One’s 300 word limit.**

Line 34: “did not apparently discriminate between sites differing in egg abundance” seems like it doesn’t gel with the preceding sentence saying females prefer previously-used sites over unexposed ones. Perhaps it might be better to say that while presence of eggs had a (qualitative) effect, there was no further (quantitative) effect of this prior clutch’s size on the focal female’s oviposition behaviour.

**We thank you for the suggestion. We have revised the sentence(s) to read on lines 27-30** “Sites that had exhibited cues produced by conspecific demonstrators were visited more, and yielded more focal offspring compared to unexposed substrates. Furthermore, patterns of bias in offspring production were consistent with ovipositing females exhibiting sensitivity to the kinship status of the prior substrate’s occupants”

Line 38: “These results [further] highlight…” might be better, as the authors already discuss some prior work on D. melanogaster as a model for studying social behaviour.

**Thank you for the suggestion. We have made that addition to the abstract. Line 33.**

Line 77: “see” = “but see”? There are a few other places where this happens, so perhaps not – but then I would just omit the “see” to have just the reference.

**We agree that the inclusion of these suggestions are unnecessary and have removed them from the revised document.**

Line 121-125: The authors now bring up the benefits of co-ovipositing with kin, but this feels a bit jumbled as the text now goes back and forth on the pros and cons of co-oviposition. Perhaps it might be better to restructure the text in the introduction to first mention benefits, then costs (or vice versa). The preceding section also goes slightly back and forth on whether they are discussing the benefits of co-laying itself, as opposed to the benefits of using social information to make decisions about whether or not to co-lay. I think this material can be shuffled around to get a more cohesive story. I do realize that these things are interconnected though, so there might not be one ideal order, but it is likely worthwhile to try something different here.

**Thank you for the restructuring suggestion. We have re-organized and reworded large portions of this paragraph to make it more cohesive and hopefully easier to follow. We begin with a brief review of evidence of social information use in oviposition site choice (Lines 83-92), why social information use may not always be the best decision (lines 93-103). We then discuss the likely reasons for the preference for laying near conspecifics (lines 103-109), the dangers involved (109-112), and how kinship may mediate these risks (lines -113-120). We feel that this is a better flow, and a simpler narrative to follow.**

Line 192-194: Can you provide details on number of generations introgressed and/or expected fraction of the genome introgressed? These results are likely in this study but ought to be mentioned here as well.

Line 196-199: Might be good to mention how many generations there have been since this divergence, instead of in years (looks to be roughly 50 generations). And while we’re at it, if you have any info on how often these have been backcrossed in that period might be nice as well.

**We have updated the methods to better explain how much back-crossing happened, and when the last round occurred (shortly prior to the start of the pandemic) (Lines 192-199).**

Line 222-227: Could you add a bit to explain the point of this procedure? I.e. to obtain kin and non-kin females from shared versus unshared environments.

**We have modified the text to make the objective of this manipulation more clear – to create all possible combinations of demonstrator females differing in the extent of relatedness and or familiarity. Lines 222-230: “**For each pair of vials, we haphazardly designated one vial as the “focal” vial, from which we collected 3 virgin females: a IV female (“the focal female”), a second IV female (the focal female’s sister, raised in the same vial), and one brown-eyed IV-*bw* female (an unrelated “familiar” female, who had developed in the same vial as the focal female). From the other (“non-focal”) vial in the pair, we collected 2 virgin females: an IV female (the focal female’s sister, raised in a different “unfamiliar” developmental environment) and one brown-eyed IV-*bw* female (an unrelated female, raised in environment that was “unfamiliar” to the focal female). In this way we were able to obtain all possible combinations of demonstrator females that differed in their genetic relatedness and their potential environental familiarity.”

Line 256: “One” = “Once”?

**Thank you for catching this typo. It has been corrected. Line 261**

Line 330-331: Can you be a bit clearer about this? I.e. what model setup is compared to what other model setup to test what effect? Same for other model comparisons.

**As part of the rewriting of the statistical we have tried to make the factors in our models more clear (see Lines 331-334, 342-345, 347-350, 358-360).**

Line 377-379: If this trend exists, show the data and/or a model; otherwise this can be omitted.

**We have decided to exclude specific reference to this trend from the text, and direct the reader to view Figure 2b. We have included an additional analysis that does show that at least 1 focal female offspring was more likely to be collected from a dish when there had been more demonstrator females (Lines 412-416)**

Line 405-407: “a dish whose demonstrator was an unrelated IV female, or a dish whose demonstrator was an unrelated IV female”; I think one of these ought to be a related IV female, right?

**You are correct. We have fixed this typo (line 449)**

Line 422: “which” = “both of which”? I would think both of those are affected by degree of kinship.

**Agreed! Line 469**

Line 428: “capable to kinship discrimination” = “capable of kinship discrimination” or “able to discriminate kin”.

**We have fixed the typo in the text to read “capable of kinship discrimination” on Line 475**

Figures: (1) The text on the axes is notably smaller than the labels indicating statistical tests. It might be nice to make these more similar (preferably by increasing the size of the text on the axes). (2) the order of brown- versus red-eyed individuals is reverted in Figure 1 relative to all other figures (which have brown-eyed first, then red-eyed).

**We have revised all the figures so that the y-axis text is larger and easier to read. It was a deliberate choice to present the red-eyed and brown-eyed figures in this way. In our first experiment, we were focused on the focal female’s behaviour, and as an afterthought wanted to highlight the competitor offspring patterns. In the subsequent experiments, our focus was on examining how changing other features or the demonstrator may (or may not) have affected the focal female, so it felt more organic to present the brown-eyed data first.**

Line 760 (Figure S1): I don’t think this is mentioned anywhere in the manuscript, but did you carry out any randomisation with how you configured this setup? Or is it always “related/familiar” top left, “unrelated/unfamiliar” top right, etc.? Not that I expect this to have any effect on the findings per se, but more that this is a detail that seemed to be missing.

**Thank you for the suggestion. The dishes themselves were always in the same position relative to each other, but the arena was randomly rotated when put down to avoid unanticipated room effects. We have added this to the manuscript on lines 255-256.**

6. PLOS authors have the option to publish the peer review history of their article (what does this mean?). If published, this will include your full peer review and any attached files.

If you choose “no”, your identity will remain anonymous but your review may still be made public.

Do you want your identity to be public for this peer review? For information about this choice, including consent withdrawal, please see our Privacy Policy.

Reviewer #1: No

Reviewer #2: No

[NOTE: If reviewer comments were submitted as an attachment file, they will be attached to this email and accessible via the submission site. Please log into your account, locate the manuscript record, and check for the action link "View Attachments". If this link does not appear, there are no attachment files.]

While revising your submission, please upload your figure files to the Preflight Analysis and Conversion Engine (PACE) digital diagnostic tool, https://pacev2.apexcovantage.com/. PACE helps ensure that figures meet PLOS requirements. To use PACE, you must first register as a user. Registration is free. Then, login and navigate to the UPLOAD tab, where you will find detailed instructions on how to use the tool. If you encounter any issues or have any questions when using PACE, please email PLOS at figures@plos.org. Please note that Supporting Information files do not need this step.

In compliance with data protection regulations, you may request that we remove your personal registration details at any time. (Remove my information/details). Please contact the publication office if you have any questions.

* ⚠ Notice: This email originated from outside of the organization. Do not click links or open attachments unless you recognize the sender and know the content is safe.
