# Supplementary material for: On the use of kinship and familiarity associated social information in mediating Drosophila melanogaster oviposition decisions
Source: PLoS One. 2025 Mar 26;20(3):e0320377. doi: 10.1371/journal.pone.0320377 (PMC11940635; doi:10.1371/journal.pone.0320377)
Supplement: Attachment — Submitted filename: Response_to_reviewers_auresp_2.docx [file pone.0320377.s008.docx]

**We would like to thank the editors and referees that have taken the time to review out manuscript. Below (in bold font) we detail how we have changed the document in response to the points raised in the last review. Line numbers referenced are those in the “clean” manuscript.**

**View Letter**

Bottom of Form

| Date: | Jan 19 2025 08:28PM |
| --- | --- |
| To: | "Emily Rakosy" emily.rakosy@mail.utoronto.ca |
| From: | "PLOS ONE" plosone@plos.org |
| Subject: | PLOS ONE Decision: Revision required [PONE-D-24-34027R1] |

PONE-D-24-34027R1

On the use of kinship and familiarity associated social information in mediating Drosophila melanogaster oviposition decisions

PLOS ONE

Dear Dr. Rakosy,

Thank you for submitting your manuscript to PLOS ONE. After careful consideration, we feel that it has merit but does not fully meet PLOS ONE’s publication criteria as it currently stands. Therefore, we invite you to submit a revised version of the manuscript that addresses the points raised during the review process.

Please ensure that you address the minor comments made by the reviewers to enhance the clarity of the manuscript and correct any typographical errors. We look forward to receiving your revised manuscript.

Please submit your revised manuscript by Mar 05 2025 11:59PM. If you will need more time than this to complete your revisions, please reply to this message or contact the journal office at [plosone@plos.org](mailto:plosone@plos.org). When you're ready to submit your revision, log on to https://www.editorialmanager.com/pone/ and select the 'Submissions Needing Revision' folder to locate your manuscript file.

Please include the following items when submitting your revised manuscript:

- A rebuttal letter that responds to each point raised by the academic editor and reviewer(s). You should upload this letter as a separate file labeled 'Response to Reviewers'.
- A marked-up copy of your manuscript that highlights changes made to the original version. You should upload this as a separate file labeled 'Revised Manuscript with Track Changes'.
- An unmarked version of your revised paper without tracked changes. You should upload this as a separate file labeled 'Manuscript'.

If you would like to make changes to your financial disclosure, please include your updated statement in your cover letter. Guidelines for resubmitting your figure files are available below the reviewer comments at the end of this letter.

If applicable, we recommend that you deposit your laboratory protocols in protocols.io to enhance the reproducibility of your results. Protocols.io assigns your protocol its own identifier (DOI) so that it can be cited independently in the future. For instructions see: <https://journals.plos.org/plosone/s/submission-guidelines#loc-laboratory-protocols>. Additionally, PLOS ONE offers an option for publishing peer-reviewed Lab Protocol articles, which describe protocols hosted on protocols.io. Read more information on sharing protocols at <https://plos.org/protocols?utm_medium=editorial-email&utm_source=authorletters&utm_campaign=protocols>.

We look forward to receiving your revised manuscript.

Kind regards,

Madhumala Sadanandappa
Academic Editor
PLOS ONE

Journal Requirements:

Please review your reference list to ensure that it is complete and correct. If you have cited papers that have been retracted, please include the rationale for doing so in the manuscript text, or remove these references and replace them with relevant current references. Any changes to the reference list should be mentioned in the rebuttal letter that accompanies your revised manuscript. If you need to cite a retracted article, indicate the article’s retracted status in the References list and also include a citation and full reference for the retraction notice.

[Note: HTML markup is below. Please do not edit.]

Reviewers' comments:

Reviewer's Responses to Questions

**Comments to the Author**

1. If the authors have adequately addressed your comments raised in a previous round of review and you feel that this manuscript is now acceptable for publication, you may indicate that here to bypass the “Comments to the Author” section, enter your conflict of interest statement in the “Confidential to Editor” section, and submit your "Accept" recommendation.

Reviewer #1: (No Response)

Reviewer #2: (No Response)

2. Is the manuscript technically sound, and do the data support the conclusions?

The manuscript must describe a technically sound piece of scientific research with data that supports the conclusions. Experiments must have been conducted rigorously, with appropriate controls, replication, and sample sizes. The conclusions must be drawn appropriately based on the data presented.

Reviewer #1: Yes

Reviewer #2: Yes

3. Has the statistical analysis been performed appropriately and rigorously?

Reviewer #1: Yes

Reviewer #2: Yes

4. Have the authors made all data underlying the findings in their manuscript fully available?

The [PLOS Data policy](http://www.plosone.org/static/policies.action#sharing) requires authors to make all data underlying the findings described in their manuscript fully available without restriction, with rare exception (please refer to the Data Availability Statement in the manuscript PDF file). The data should be provided as part of the manuscript or its supporting information, or deposited to a public repository. For example, in addition to summary statistics, the data points behind means, medians and variance measures should be available. If there are restrictions on publicly sharing data—e.g. participant privacy or use of data from a third party—those must be specified.

Reviewer #1: Yes

Reviewer #2: Yes

5. Is the manuscript presented in an intelligible fashion and written in standard English?

PLOS ONE does not copyedit accepted manuscripts, so the language in submitted articles must be clear, correct, and unambiguous. Any typographical or grammatical errors should be corrected at revision, so please note any specific errors here.

Reviewer #1: Yes

Reviewer #2: Yes

6. Review Comments to the Author

Please use the space provided to explain your answers to the questions above. You may also include additional comments for the author, including concerns about dual publication, research ethics, or publication ethics. (Please upload your review as an attachment if it exceeds 20,000 characters)

Reviewer #1: Thanks to the authors for incorporating suggestions in their revised manuscript – I am happy my comments have been addressed, except this one point:

I’m afraid the edits to lines 457-463 (now 507-15…or should that be 539-48? I think many of the line numbers given in the response letter are incorrect) haven’t really helped me, and there are some sentence structure problems. Could the passage be summarised more concisely? e.g. along the lines of:

“A previous study found that D. melanogaster chose to oviposit near eggs of other females whose developmental diet matched their own, regardless of relatedness or the composition of the diet (Malek and Long, 2020).”

**We are very pleased to read that the reviewer is (almost) completely satisfied with the changes to the document, and hope that the changes below to line 506-509 (on the clean document) is satisfactory:**

**A previous study found that *D. melanogaster* females were more likely to oviposit near eggs laid by other females whose developmental diet matched their own, regardless of their population of origin or the composition of the diet itself (Malek and Long 2020).**

Reviewer #2: Dear editor,

I have read the revised version of manuscript PONE D-24-34027, “On the use of kinship and familiarity associated social information in mediating Drosophila melanogaster oviposition decisions” by Emily Rakosy, Sanduni Talagala, and Tristan A.F. Long, along with their replies to the queries raised during the first round of review. Altogether, I think the authors have substantially improved their manuscript. By and large, they have managed to either make edits that ameliorate any issues raised, or have clarified their reasoning for sticking to their original work; thank you for these efforts!

**We thank you for your kind words. We are very happy with the revised manuscript: it has benefitted from the rigorous previous review, and are grateful for the opportunity to share this work with the readership of PLoS ONE.**

I only have some minor recommendations on e.g. typos (probably some still remain) and some phrases that I thought were unclear.

**We appreciate your eye for detail. In addition to correcting the text in the manner described below we have also sone a thorough re-reading of the manuscript to pick up any other typographical or grammatical errors.**

Minor comments:
Line 14: “Decisions” = Deciding”?

**The sentence has been changed to “Information produced by conspecifics can potentially be useful in making decisions as this “social information” may provide an energetically cheaper means of assessing oviposition site suitability rather than acquiring it personally.” (Lines 14-17)**
Line 40: I think several of these keywords can be omitted as they are also in the title (Drosophila melanogaster, oviposition).

**We have removed the redundant keywords (Lines 37-38)**
Line 97: “… Dukas, 2009 and Battesti…” = “… Dukas, 2009, Battesti…”?

**We have fixed the typo on line 92: “ovipositing (Sarin and Dukas 2009, Battesti *et al.* 2012), the presence”**
Line 164: “might not provide” = “might provide”? This doesn’t seem logical otherwise.

**Thank you for catching that error. The “not” has been removed (line 154)**
Line 170: “investing into mates” = “investing into mating”?

**We have rephrased the sentence (line 158-160) to read: “Lizé et al. (2014) described how a male fruit fly’s ability to strategically avoid mating with a sibling was impeded if they had both developed in the same environment.”**
Line 439-440: “Interestingly, dishes where there had been two demonstrator females has been present were significantly…”; I don’t know what this is supposed to read exactly, but I think the “has been present” bit can be dropped.

**We have rephrased this sentence (line 413-416) to read “Furthermore, dishes that had been exposed to two demonstrator females were significantly *less* likely to yield *zero* focal female offspring (8/48) than those dishes that had been exposed to either one demonstrator female (25/48) or from control dishes (32/48; χ^2^ =13.43, df=2, p=0.0012).” which hopefully emphasizes that focal females are more likely to produce offspring when there are more conspecific (brown-eyed) eggs present – contrary to what we would have expected if they were avoiding the higher-density patches. We hope that this reads better.**
Line 468: Period after statistical results should be a comma?

**Thank you. This has been fixed (line 439)**
Line 520: “non kin” = “non-kin”?

**Thank you. This has been changed (line 489)**
Line 522: “trh” = “in the”?

**Thank you. This has been changed (line 491)**
Line 524: “females are selectively use” = “females selectively use”? If so, also edit “favouring” on line 525 to “favour”.

**We have changed the sentence (line 493-495) to read “These results suggest that ovipositing females are selective in their use of social information, and favor cues produced by their relatives, while perceived environmental familiarity does not enhance oviposition site use.”**

Line 527: I recommend to separate the last bit from “as demonstrator…” onward. That way you can explain how the transfer of gut bacteria etc. by females affects olfactory cues, which can be affected by other components to distort the information conferred by these cues.
Line 535-536: I had to read this a few times to get it; I think removing the commas around “and potentially misleading” might make this clearer. In fact, this sentence is a bit of a beast anyway, so maybe some general editing/splitting here might be helpful (this sentence goes all the way from line 533 to 538…).

**As suggested, we have broken up this sentence and rewritten it for clarity (lines 495-499): “Determining the specific cues being used by focal females is beyond the scope of this study as there are numerous ways that social information may be obtained. When ovipositing, female fruit flies transfer gut bacteria, CHCs, and male-specific compounds acquired during copulation to the surface of their eggs and the media (Bakula 1969, Guilhot et al. 2023, Moreira-Soto et al. 2024).”**
Line 555: “rule-out” = “rule out”?

**Thank you. This has been changed (line 516)**
Line 574: “experiment one” = “experiment 1”? (for more consistency in style).

**Thank you. This has been changed (line 533)**

7. PLOS authors have the option to publish the peer review history of their article ([what does this mean?](https://journals.plos.org/plosone/s/editorial-and-peer-review-process#loc-peer-review-history)). If published, this will include your full peer review and any attached files.


If you choose “no”, your identity will remain anonymous but your review may still be made public.


**Do you want your identity to be public for this peer review?** For information about this choice, including consent withdrawal, please see our [Privacy Policy](https://www.plos.org/privacy-policy).

Reviewer #1: No

Reviewer #2: No

[NOTE: If reviewer comments were submitted as an attachment file, they will be attached to this email and accessible via the submission site. Please log into your account, locate the manuscript record, and check for the action link "View Attachments". If this link does not appear, there are no attachment files.]

While revising your submission, please upload your figure files to the Preflight Analysis and Conversion Engine (PACE) digital diagnostic tool, <https://pacev2.apexcovantage.com/>. PACE helps ensure that figures meet PLOS requirements. To use PACE, you must first register as a user. Registration is free. Then, login and navigate to the UPLOAD tab, where you will find detailed instructions on how to use the tool. If you encounter any issues or have any questions when using PACE, please email PLOS at [figures@plos.org](mailto:figures@plos.org). Please note that Supporting Information files do not need this step.

*In compliance with data protection regulations, you may request that we remove your personal registration details at any time.* [*(Remove my information/details)*](https://www.editorialmanager.com/pone/login.asp?a=r)*. Please contact the publication office if you have any questions.*

Top of Form

Bottom of Form
